# A computational analysis of retinal image quality in eyes with keratoconus

**DOI:** 10.1038/s41598-020-57993-w

**Published:** 2020-01-28

**Authors:** Vinay Kumar Nilagiri, Sangeetha Metlapally, Clifton M. Schor, Shrikant R. Bharadwaj

**Affiliations:** 1 Brien Holden Institute of Optometry and Vision Sciences, L V Prasad Eye Institute, Road no. 2, Banjara Hills, Hyderabad, 500034 Telangana India; 2 Prof. Brien Holden Eye Research Centre, Hyderabad Eye Research Foundation, L V Prasad Eye Institute, Road no. 2, Banjara Hills, Hyderabad, 500034 Telangana India; 3 Vision Science Group, University of California Berkeley School of Optometry, Berkeley, USA

**Keywords:** Preclinical research, Translational research

## Abstract

Higher-order aberrations (HOA’s) are exaggerated in eyes with keratoconus but little is known about their impact on the retinal image quality (IQ) of these eyes. This computational study determined changes in IQ [peak IQ, best focus and depth of focus (DOF)] of 12 subjects with manifest keratoconus in both eyes (KCE cohort), 9 subjects with very asymmetric ectasia (VAE cohort) with and without their Rigid Gas Permeable contact lenses (RGP CL’s) and 20 age-matched controls, using a HOA-based through-focus analysis performed on the logNS IQ metric over 5 mm pupil diameter following cycloplegia. All IQ parameters were significantly worse in the KCE cohort with their native HOA’s, relative to controls and in the ectatic eye of the VAE cohort, relative to the fellow non-ectatic eye (p ≤ 0.008 for all). Reduction in HOA’s of these eyes with RGP CL’s resulted in a significant improvement in all IQ parameters but they all remained significantly poorer than controls (p ≤ 0.02 for all). The inter-subject variability of best focus and the DOF range were inversely related to peak IQ in these eyes (r = 0.85; p < 0.001). These results provide the optical basis for two clinical observations on keratoconus: (1) optical performance of keratoconic eyes are significantly better with RGP CL’s than with spectacles or unaided conditions and (2) the endpoint of subjective refraction is elusive in keratoconic eyes, relative to healthy controls or to the non-ectatic eye in bilaterally asymmetric ectasia.

## Introduction

Keratoconus is a progressive, non-inflammatory disease of one or both eyes characterized by thinning, anterior protrusion, increased asphericity and an eventual scarring and opacity of the cornea^1^. The disease may manifest itself with similar or dissimilar severity in the two eyes^1^. Optically, the disease is characterized by increased magnitude of sphero-cylindrical refractive errors and higher-order aberrations (HOA’s)^2–5^. Many correction modalities including spectacles, soft toric, rigid gas permeable (RGP) and scleral CL’s (CL) that reduce corneal asphericity are currently available for improving visual performance of these eyes^1^. Specifically, spatial and depth-vision (e.g. logMAR and stereoacuity) of keratoconic eyes has been shown to improve with RGP CL wear, relative to sphero-cylindrical spectacles^6–9^. This improvement is further enhanced by correcting these eyes with customized wavefront correcting CL’s or lab-based adaptive optics apparatus^10–13^.

Despite these advances, there is little information on the underlying changes in retinal image quality (IQ) of these eyes computed from objective wavefront measurements. A systematic analysis of IQ provides important insights into the performance of a keratoconic eye as an optical system, which determines the quality of information available for neural processing that ultimately governs visual performance. IQ significantly deteriorates in the presence of lower-and higher-order aberrations of the eye^14–18^. The loss of IQ in keratoconus with uncorrected lower-order aberrations (i.e. defocus and astigmatism) and its improvement with optical correction is well known and is a part of the routine clinical management of the disease^4,6,8^. This study aimed at systematically describing the impact of higher-order aberrations (HOA’s) and its reduction with RGP CL’s on the IQ of keratoconic eyes. This study also used the IQ analysis to provide the optical basis for two common clinical observations in keratoconus: i) the visual performance of keratoconic eyes improves significantly from spectacles to RGP CL wear and ii) the endpoint of subjective refraction is very elusive in these eyes, more so with spectacles than with RGP CL’s, all relative to those without keratoconus. To achieve these goals, through-focus curves were constructed computationally from the subject’s wavefront aberration map and the following three IQ parameters were derived: the peak IQ achieved, dioptric location of this peak IQ (best focus) and the sensitivity of the optical system to changes in dioptric foci [i.e. the depth of focus (DOF)]^16,18^. Clinically, these parameters translate into best spatial resolution acuity, dioptric endpoint of subjective refraction that yields best acuity and sensitivity of the visual system to changes in optical blur, respectively.

Previous studies investigating the impact of HOA’s on IQ either by inducing them on otherwise healthy eyes^17–21^ or by investigating this relationship in subjects with corneal distortions due to disease or iatrogeny (e.g. LASER refractive surgery^16,22^) have shown a systematic loss of IQ and a persistence of this sub-optimal IQ over a larger dioptric range in the presence of increased HOA’s^16–22^. The dioptric location of best focus may also shift significantly away from emmetropia in these eyes depending on the magnitude and type of HOA’s^16–18,23^. Similar trends may also be predicted in keratoconic eyes given the increased HOA’s^2–5^, vis-à-vis, age-matched controls.

## Results

A total of 12 subjects with manifest keratoconus in both eyes, 9 subjects with very asymmetric ectasia and 20 age-matched controls [20.5 yrs (20–21 yrs)] participated in the study. Tables 1–3 provide the demographic details and the high-contrast logMAR visual acuity of the participants in both cohorts of this study.

**Table 1:** Demographic and clinical details of the subjects in the KCE and VAE cohorts.

Subject number	Age (yrs)	Gender	Age of onset (yrs)	Ectatic eye(s)	Habitual optical correction	CCT right eye (μ)	CCT lefteye (μ)	Steep K righteye (D)	Steep K left eye (D)
**KCE cohort**									
1	31	M	NA	BE	RGP CL	392	409	56.6	50.6
2	22	F	NA	BE	RGP CL	488	484	47.4	51.5
3	19	M	NA	BE	RGP CL	415	438	57.8	54.3
4	20	M	NA	BE	RGP CL + OS CXL	NA	NA	NA	NA
5	19	M	NA	BE	RGP CL	NA	NA	NA	NA
6	20	M	12	BE	RGP CL	391	437	61.7	55.4
7	18	F	15	BE	RGP CL	390	427	57.7	50.7
8	18	M	8	BE	RGP CL	478	465	52.2	54.7
9	18	F	NA	BE	RGP CL	462	461	49.4	54.2
10	18	M	NA	BE	RGP CL	385	451	57.3	47.7
11	18	F	24	BE	RGP CL	443	446	47	48.7
12	19	F	NA	BE	RGP CL	541	544	45.6	43.8
Median (IQR)	19 (18–20)					429 (391–474)	448.5 (437–464)	54.4 (47–57)	51.1 (49–54)
**Subject number**	**Age (yrs)**	**Gender**	**Age of onset (yrs)**	**Ectatic eye**	**Habitual optical correction**	**CCT ectatic eye (μ)**	**CCT non-ectatic eye (μ)**	**Steep K ectatic eye (D)**	**Steep K non-ectatic eye (D)**
**VAE cohort**									
1	18	M	18	RE	RGP CL	514	519	54.8	43.3
2	19	F	20	LE	RGP CL	498	513	48.9	46.1
3	23	F	NA	LE	RGP CL	485	516	50.2	44.2
4	26	M	NA	LE	RGP CL + OS CXL	493	527	48.7	44.7
5	18	F	16	LE	RGP CL	533	554	51.3	47.2
6	18	M	15	RE	RGP CL	382	457	52.9	45.2
7	21	M	21	RE	RGP CL + OD CXL	492	521	52.8	46
8	18	M	15	RE	RGP CL + OD CXL	420	462	52.9	42.6
9	25	M	25	RE	RGP CL	409	463	56.6	45.1
Median (IQR)	19 (18–23)					492 (420–498)	516 (463–521)	52.8 (50–52)	45.1 (44–46)

The age of onset of keratoconus is self reported by the patient. CXL indicates collagen crosslinking procedure performed on the subject as a part of the disease management. Central corneal thickness (CCT) and steep values of keratometry (Steep K) are obtained from standard topography devices as a part of the patient’s examination (The WaveLight® Oculyzer™, Alcon, USA). NA indicates data was not available on that parameter in the subject.

**Table 2:** High-contrast logMAR acuity of subjects in the KCE cohort and VAE cohort under unaided, spectacle-corrected and RGP CL-corrected viewing conditions.

Subject number	Unaided viewing		Spectacle corrected viewing		RGP CL corrected viewing	
**KCE cohort**						
	**Right eye**	**Left eye**	**Right eye**	**Left eye**	**Right** **eye**	**Left eye**
1	1.06	0.62	0.38	0.16	0.10	0.08
2	0.44	0.54	−0.08	0.32	−0.10	0.02
3	0.70	0.40	0.30	0.18	0.10	0.00
4	0.22	0.86	0.20	0.28	0.14	0.06
5	0.60	0.20	0.20	0.10	0.12	0.00
6	1.12	0.26	0.28	0.34	0.12	−0.02
7	0.86	0.50	0.40	0.08	0.00	−0.02
8	0.96	0.90	0.18	0.10	−0.06	0.00
9	0.22	0.42	0.04	0.12	0.04	0.00
10	0.44	0.14	0.56	0.02	0.04	−0.10
11	0.30	0.34	0.10	0.06	0.00	0.00
12	0.90	0.56	0.70	0.24	0.18	0.10
Median (IQR)	0.65 (0.41–0.92)	0.46 (0.32–0.58)	0.24 (0.16–0.39)	0.14 (0.10–0.25)	0.07 (0.00–0.12)	0.00 (−0.01–0.03)
**VAE cohort**						
	**Ectatic eye**	**Non-ectatic eye**	**Ectatic eye**	**Non-ectatic eye**	**Ectatic eye**	**Non-ectatic eye**
1	0.22	−0.08	0.18	0.04	0.02	—
2	0.52	0.52	0.48	0.02	0.14	—
3	0.44	0.02	0.48	−0.06	0.00	—
4	0.52	0.42	0.06	−0.04	0.02	—
5	1.16	0.06	0.40	−0.06	0.00	—
6	0.46	0.02	0.20	−0.08	0.04	—
7	0.60	0.36	0.12	−0.06	0.04	—
8	0.58	0.30	0.22	0.00	0.06	—
9	0.22	0.02	0.00	−0.12	0.04	—
Median (IQR)	0.52 (0.44–0.58)	0.06 (0.02–0.36)	0.20 (0.12–0.40)	−0.06 (−0.06–0.00)	0.04 (0.02–0.04)	—

LogMAR acuity was obtained as a part of patient’s routine eye examination using a computerized acuity measurement system (Complog®, Clinical Vision Measurement Systems Ltd, UK)^40^.

**Table 3:** Median (25^th^–75^th^ IQR) right eye, left eye, interocular average and interocular difference of the RMS deviation of higher-order aberrations (HORMS) of subjects in the KCE and VAE cohorts under unaided and RGP contact lens wearing conditions and those of controls under the best-corrected conditions.

	Right eye of controls or KCE cohort(µ)	Left eye of controls or KCE cohort (µ)	Interocular average (µ)	Interocular difference (µ)
**Controls**				
HORMS – Unaided	0.35(0.27–0.42)	0.36(0.24–0.46)	0.37(0.24–0.46)	0.06(0.03–0.13)
**KCE cohort**				
HORMS – Unaided	1.96(1.50–2.44)	1.57(1.38–2.25)	1.69(1.46–2.08)	0.62(−0.43–1.10)
HORMS – RGP CL	0.62(0.49–0.83)	0.60(0.48–0.63)	0.62(0.52–0.80)	0.09(−0.04–0.25)
	**Ectatic eye of VAE cohort (µ)**	**Non-ectatic eye of VAE cohort (µ)**	**Interocular average (µ)**	**Interocular difference (µ)**
HORMS – Unaided	1.76(1.36–2.35)	0.52(0.47–0.63)	1.20(0.89–1.49)	1.15(1.05–1.72)
HORMS – RGP CL	0.78(0.61–0.94)	–	0.61(0.55–0.71)	0.27(0.03–0.49)

The pattern of the RMS deviations of all HOA’s (HORMS) and some key higher-order Zernike coefficients under optically unaided viewing conditions and their reduction with RGP CL’s are shown in Table 4 and 5 for the KCE and VAE cohorts. The median HORMS of the right and left eye, the interocular average and the interocular difference in HORMS of the KCE cohort decreased significantly from unaided to RGP CL conditions (Wilcoxon sign rank test; Z ≥ 3.7; n = 12; p ≤ 0.002) (Table 3). For the VAE cohort, the HORMS of the ectatic eye decreased from unaided to RGP CL wear but remained larger than the HORMS of the fellow eye without ectasia (Table 3). The median interocular average and difference in HORMS of the VAE cohort also decreased with RGP CL wear, relative to optically unaided viewing conditions (Table 3). All the aforementioned changes in HORMS from unaided to RGP CL wear were statistically significant in the VAE cohort (Z ≥ 35; n = 9; p ≤ 0.001). All HORMS values of controls were significantly different from both the KCE cohort and the ectaticeye of the VAE cohort (Kruskal Wallis test; H = 21.9, df = 2; p < 0.0001) (Table 3).

**Table 4:** Median (25^th^–75^th^ IQR) coefficients of key higher-order Zernike coefficients of the two eyes under unaided and RGP CL wearing conditions in KCE cohort, VAE cohort and controls under the best-corrected conditions.

	Unaided right eye of controls or KCE cohort (µ)	Unaided left eye of controls or KCE cohort (µ)	RGP CL right eye of KCE cohort (µ)	RGP CL left eye of KCE cohort (µ)
**Controls**				
Trefoil; Z(3, −3)	−0.08 (−0.14–0.02)	−0.05 (−0.10–0.03)	—	—
Vert coma; Z(3, −1)	−0.13 (−0.21–0.01)	−0.10 (−0.19–0.02)	—	—
Horiz coma; Z(3, 1)	0.15 (0.04–0.21)	0.10 (−0.003–0.27)	—	—
Trefoil; Z(3, 3)	0.03 (−0.05–0.09)	−0.03 (−0.08–0.03)	—	—
Sph aberration; Z(4, 0)	0.07 (−0.04–0.16)	0.05 (−0.01–0.19)	—	—
**KCE cohort**				
Trefoil; Z(3, −3)	0.64 (0.32–0.98)	0.53 (0.21–0.93)	0.00 (−0.04–0.04)	0.01 (0.00–0.06)
Vert coma; Z(3, −1)	−0.96 (−1.20–−0.69)	−1.27 (−1.94–−0.52)	0.20 (−0.07–0.42)	0.19 (0.02–0.35)
Horiz coma; Z(3, 1)	−0.67 (−1.31–−0.49)	−0.19 (−0.41–−0.10)	0.35 (0.16–0.44)	0.12 (0.03–0.29)
Trefoil; Z(3, 3)	0.13 (−0.02–0.44)	0.20 (0.03–0.48)	−0.06 (−0.12–−0.03)	−0.01 (−0.06–0.03)
Sph aberration; Z(4, 0)	−0.40 (−0.46–−0.08)	−0.31 (−0.46–−0.05)	0.29 (0.21–0.37)	0.32 (0.24–0.36)
	**Unaided ectatic eye of VAE cohort (µ)**	**Unaided non-ectatic eye of VAE cohort (µ)**	**RGP CL ectatic eye of VAE cohort (µ)**	**RGP CL non-ectatic eye of VAE cohort (µ)**
Trefoil; Z(3, −3)	0.29 (0.08–0.87)	0.07 (−0.04–0.25)	−0.09 (−0.14–0.07)	—
Vert coma; Z(3, −1)	−1.30 (−1.56–−0.84)	−0.27 (−0.43–−0.18)	0.36 (0.30–0.48)	—
Horiz coma; Z(3, 1)	−0.52 (−1.14–−0.01)	0.14 (0.06–0.21)	0.24 (0.19–0.34)	—
Trefoil; Z(3, 3)	0.07 (−0.18–0.43)	−0.11 (−0.18 –−0.01)	−0.02 (−0.09–0.08)	—
Sph aberration; Z(4, 0)	−0.46 (−0.76–−0.28)	−0.06 (−0.10–0.02)	0.36 (0.31–0.39)	—

With regards to the individual Zernike terms, the coefficients of vertical coma [Z(3, −1)], horizontal coma [Z(3, 1)] and spherical aberration [Z(4, 0)] showed the most negative values in both KCE cohort and in the ectatic eye of the VAE cohort under unaided conditions and all of these terms decreased to small positive values after RGP CL wear (KCE cohort: |Z| ≥ 2.10; n = 12; p ≤ 0.03; ectatic eye of VAE cohort: |Z| ≥ 2.07; n = 9; p ≤ 0.05) (Table 4). The two trefoil terms [Z(3, −3) and Z(3, 3)] showed a relatively large positive value under unaided conditions and they were decreased to insignificant levels after RGP CL wear in both cohorts (KCE cohort: |Z| ≥ 1.43; n = 12; p ≤ 0.05; ectatic eye of VAE cohort: |Z| ≥ 0.84; n = 9; p ≤ 0.05) (Table 4). All Zernike terms higher than the 4^th^ order were of insignificant magnitudes for both viewing conditions in both cohorts. As expected, the unaided HOA terms of controls and that of the non-ectatic eyes of the VAE cohort were in general smaller than those of the ectatic eyes under unaided and RGP CL conditions (|H| ≥ 18.6; df = 2; p < 0.001) (Table 4).

**Figure 1:**
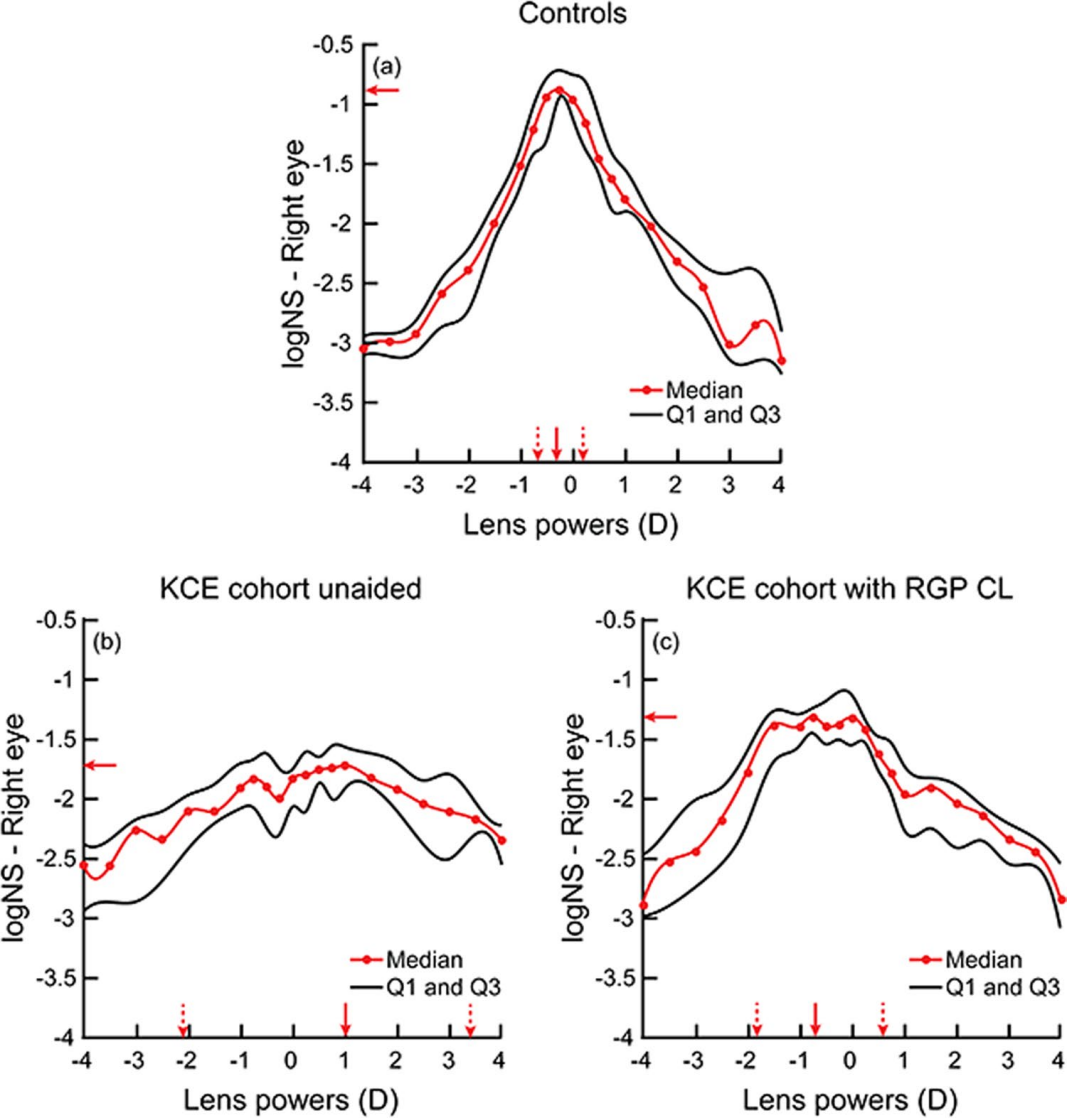
Median (25^th^–75^th^ IQR) through-focus curves of controls (n = 20) (**a**), right eye of KCE cohort (n = 12) under unaided viewing (**b**) and with RGP contact lens (**c**) obtained by plotting logNS for each value of induced myopia and hyperopia. In this figure and in Fig. 3, induced myopia is indicated as positive numbers while induced hyperopia is indicated as negative numbers along the abscissa. The solid circles indicate individual data points while the curve indicates interpolated data. The lower and the upper thin curves indicate 25th and 75th interquartile range of the through-focus curve, respectively. Horizontal arrow indicates peak IQ, solid and dashed vertical arrows indicate best focus and depth of focus range, respectively.

Figure 1 shows the median (25^th^–75^th^ IQR) through-focus curves of the right eyes of controls (panel A) and KCE cohort without any correction (panel B) and with their RGP CL correction (panel C). Data from the left eyes of both cohorts were very similar to the respective right eyes and they will henceforth not be shown separately. Qualitatively, the through-focus curve of controls showed the characteristic inverted V-pattern with the peak IQ achieved close to emmetropia followed by a monotonic drop in IQ with increasing values of myopic and hyperopic defocus (increasingly negative values of logNS indicates worsening of IQ) (Fig. 1a). In comparison, the through-focus curves generated for the unaided eyes of the KCE cohort did not have a distinctive best focus position and had gradually changing IQ across the entire range of myopic and hyperopic defocus (Fig. 1b). When these eyes were corrected with RGP CL’s, the through-focus curve now had a more distinctive peak in IQ and best focus position with monotonic drop in IQ with induced myopic and hyperopic defocus (Fig. 1c).

**Figure 2:**
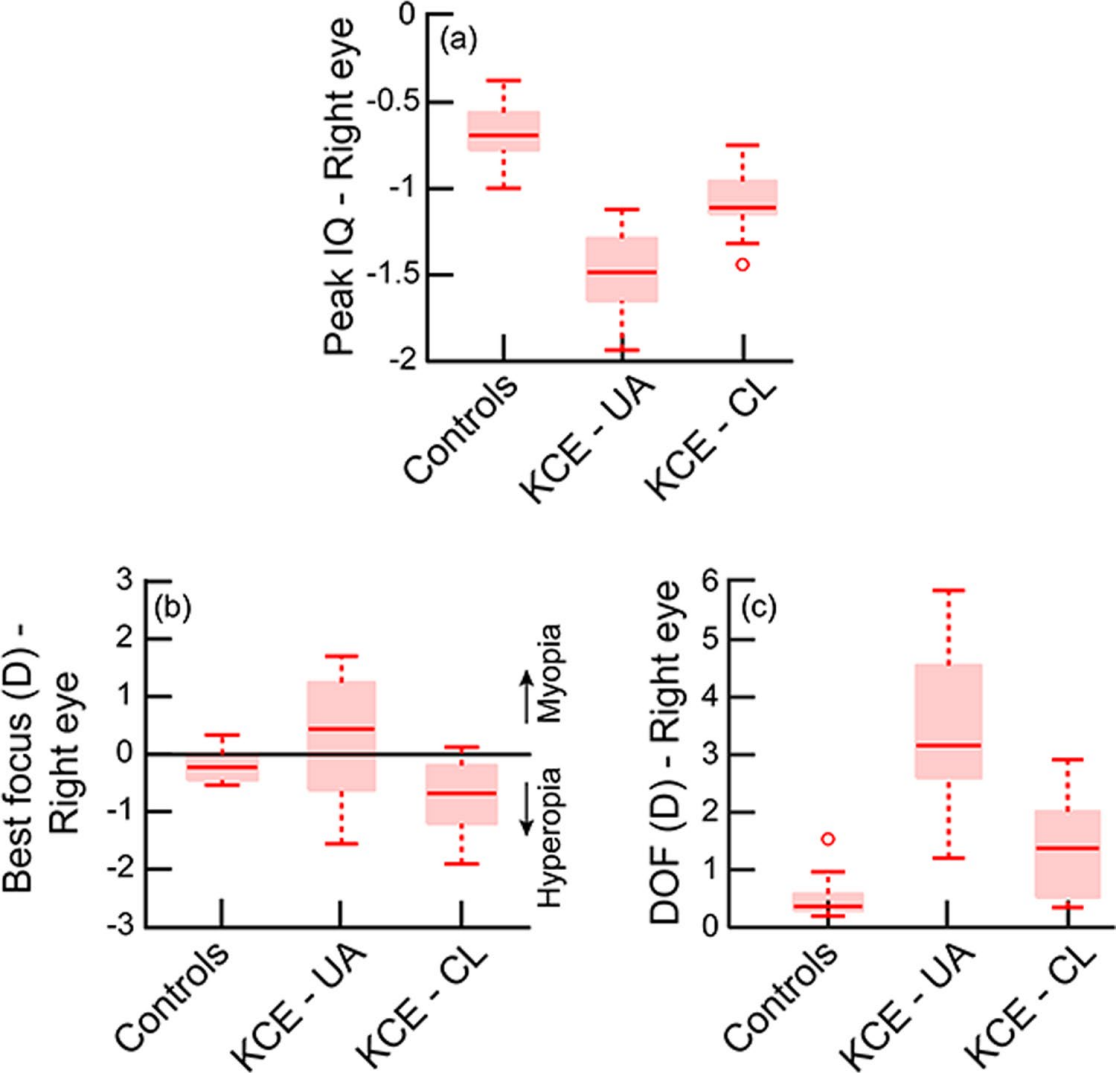
Box and Whisker plots of the peak IQ (**a**), best focus (**b**) and DOF (**c**) of controls and unaided and RGP contact lens viewing of the KCE cohort. The solid horizontal line within the box indicates the group median, lower and upper edges of the box indicate the 25^th^ and 75^th^ interquartile range (IQR) and lower and upper whiskers show the 1st and 99th quartiles. The horizontal line in panel B through zero diopters indicates optical infinity. Only data from the right eye of the KCE cohort is shown in this figure for clarity.

The median peak IQ and the DOF of the KCE cohort with RGP CL wear were statistically significantly different from the unaided conditions (peak IQ: W = −66; Z = −2.91; p = 0.04; DOF: W = 64; Z = 2.82; p = 0.004) but they did not quite reach the level of the control cohort (Mann Whitney U test; peak IQ: U = 228.5; Z = −4.2; p < 0.001; DOF: U = 40.5; Z = 3.08; p < 0.002) (Figs. 1, 2a,c). The median best focus of the KCE cohort shifted from a myopic defocus value to a small hyperopic defocus value when switching from unaided to RGP contact lens wearing conditions (Fig. 1). The median best focus of the KCE cohort were significantly different from that of controls for both unaided and RGP CL conditions (H = 10.4; df = 2; p = 0.006) (Fig. 2b). The inter-subject variability of the through-focus curves of the KCE cohort also decreased from unaided to RGP CL wearing conditions but it did not quite reach the level of the control cohort (Fig. 1).

**Figure 3:**
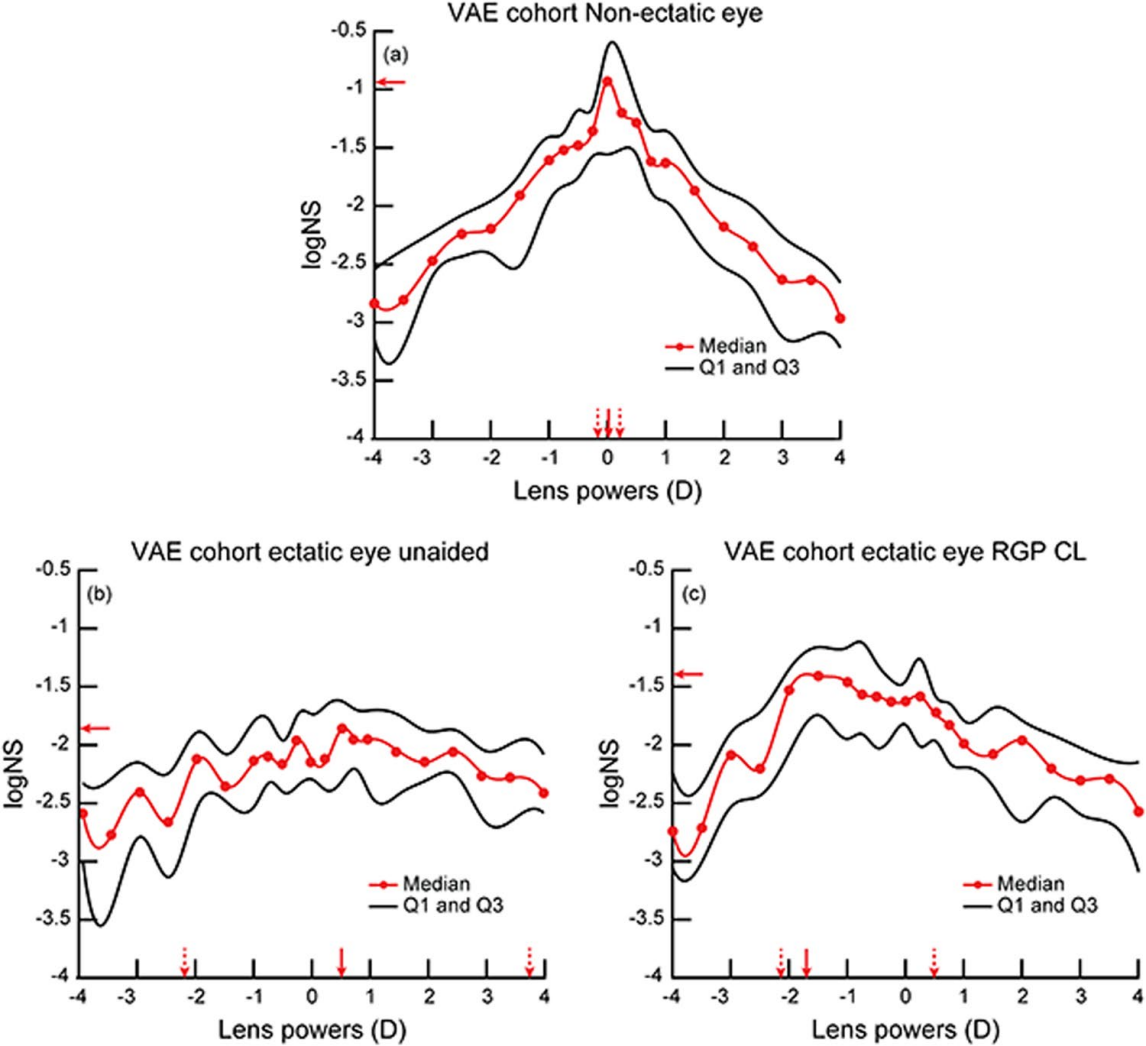
Median (25^th^–75^th^ IQR) through-focus curves of non-ectatic eye (**a**), ectatic eye under unaided viewing (**b**) and ectatic eye under RGP contact lens viewing of the VAE cohort (n = 9) (**c**). All other details are similar to Fig. 1.

Figure 3a–c plot the median (25^th^–75^th^ IQR) through-focus curves of non-ectatic eye (panel A), ectatic eye under unaided viewing (panel B) and ectatic eye with RGP CL’s (panel C) in the VAE cohort. In general, the median through-focus curves of the ectatic eye of the VAE cohort under unaided conditions resembled the KCE cohort while the through-focus curves of the non-ectatic eye of the VAE cohort resembled the control cohort (Fig. 3a,b).

**Figure 4:**
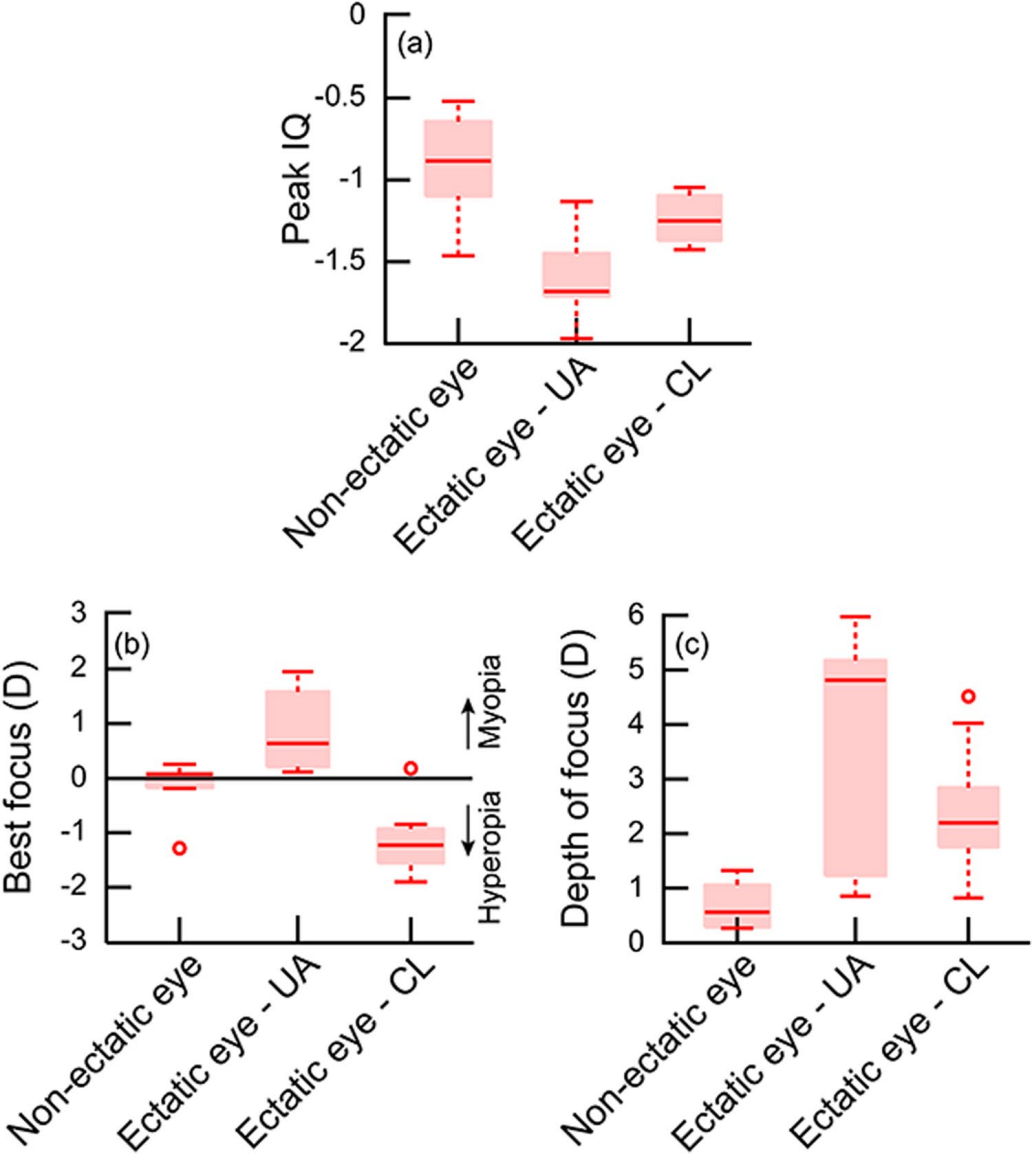
Box and Whisker plots of the peak IQ (**a**), best focus (**b**) and DOF (**c**) of the non-ectatic eye, ectatic eye under unaided viewing and ectatic eye under RGP contact lens viewing of the VAE cohort. All other details are similar to Fig. 2.

The median unaided peak IQ and DOF of the ectatic eye was statistically significantly poorer and wider, respectively, than the data from the same eye with RGP CL correction and from the data of the fellow non-ectatic eye (peak IQ: W = 2; Z = −2.02; p ≤ 0.05; DOF: W = 9; Z = −0.84; p ≤ 0.05) (Fig. 4a,c). The peak IQ and DOF of the RGP CL corrected ectatic eye remained significantly different from that of the fellow non-ectatic eye, suggesting that the improvement in optical quality of the RGP CL corrected ectatic eye did not reach up to the level of the non-ectatic eye (Fig. 4a,c). The median best focus of the ectatic eye of the VAE cohort was significantly different to the non-ectatic eye under both unaided and RGP CL conditions (H = 16.48; df = 2; p = 0.0003). All three IQ parameters of the non-ectatic eye in the VAE cohort were not significantly different from the right eye of the control cohort (U = 203, Z = 0.43, p = 0.67).

**Figure 5:**
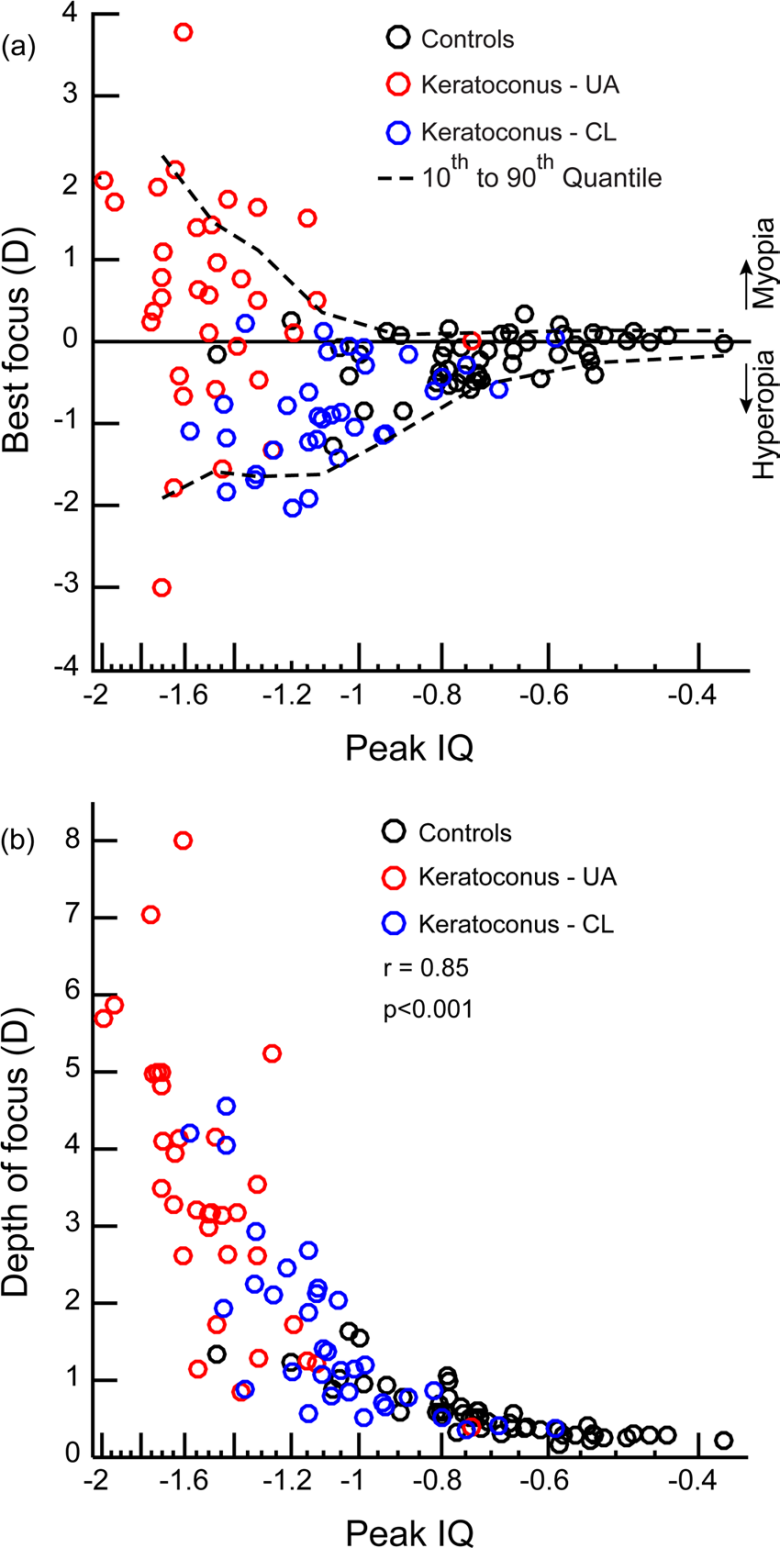
Scatter diagram showing the relation between best focus and peak IQ (**a**) and depth of focus and peak IQ (**b**) across all cases (both KCE and VAE cohorts) and controls that participated in this study. The solid horizontal line in panel A though zero diopters indicates optical infinity and the dashed curves above and below the solid horizontal line indicates the 10^th^ and 90^th^ quantiles of best focus data. The converging trend of the upper and lower quantile curves with improving peak IQ indicates a reduction in the inter-subject variability of best focus with improving image quality.

Figure 5 plots the relation between peak IQ and best focus (panel A) and between peak IQ and DOF (panel B) for all cases (both KCE and VAE cohorts) and controls that participated in this study. Even while best focus was poorly correlated with peak IQ (Spearman’s correlation coefficient r = 0.19; p = 0.63), there was a clear trend for the inter-subject variability of best focus to increase with a loss in peak IQ (Fig. 5a). This trend was evident from the widening range of the 10^th^–90^th^ IQR with a loss in peak IQ (Fig. 5a). The DOF, on the other hand, was well correlated with peak IQ across all subjects that participated in the study, indicating a progressive widening of the DOF with a loss in peak IQ (r = 0.85; p < 0.001) (Fig. 5b). The correlation coefficients and the corresponding p-value for the individual cohorts are as follows: controls (r = −0.80; p < 0.001), unaided KCE cohort (r = −0.69; p = 0.003), RGP CL corrected KCE cohort (r = −0.74; p < 0.001), unaided ectatic eye of VAE cohort (r = −0.82; p < 0.001), RGP CL corrected ectatic eye of VAE cohort (r = −0.60; p = 0.08), unaided non-ectatic eye of VAE cohort(r = −0.97; p < 0.001).

## Discussion

### The results of the study can be summarized as follows

(i) As observed in previous studies^2–5^, the HOA’s of keratoconic eyes reduced with RGP CL’s relative to unaided conditions, but remained significantly higher than those of controls (Tables 3 and 4).
(ii) The peak IQ was lower and the DOF was wider in keratoconic eyes under unaided viewing conditions, relative to RGP CL wear (Figs. 1–4). While the overall optical quality of these eyes improved with RGP CL’s, all three IQ parameters remained poorer than that of controls (Figs. 1–4, Tables 3 and 4).
(iii) The best focus of ectatic keratoconic eyes shifted from a myopic endpoint with their native HOA’s to a hyperopic endpoint when HOA’s were minimized with RGP CL’s. The best focus, however, remained significantly different from the emmetropic endpoint, as observed in controls (Figs. 2 and 4).
(iv) The IQ of the ectatic eye of the VAE cohort was similar to the KCE cohort while that of the non-ectatic eye resembled the controls (Figs. 3 and 4).
(v) Across all subjects with keratoconus and controls that participated in the study, a loss in peak IQ was associated with a widening of DOF and an increase in inter-subject variability of best focus (Fig. 5).

Visual performance of keratoconic eyes is better with RGP CL’s than spectacles, but they remain poorer than that of controls^8,12^. The present study provides the optical basis for these results by showing that the peak IQ of keratoconic eyes improves with RGP CL, relative to unaided conditions, but they continue to remain poorer than that of controls (Tables 3 and 4, Figs. 1–4). The extent of change in IQ in these eyes most likely arises from the magnitude of HOA’s that were corrected with RGP CL’s, relative to unaided conditions, and how much remained, relative to controls (Tables 3 and 4)^4^. In the present cohort, the unaided HORMS of eyes with keratoconic ectasia was approximately five-fold larger than that of controls and this difference reduced to about two-fold with RGP CL’s (Table 3). Further reductions in the eye’s HOA’s, for instance, with custom-designed wavefront optimized CL’s, may improve the peak IQ of these ectatic eyes even further than what is reported here^5,10,11^. Interestingly, Sabesan and Yoon reported that the monocular logMAR acuities of keratoconic eyes remained poorer than controls even after full correction of the eye’s wavefront aberrations using an adaptive optics system^12^. These results imply that keratoconics may experience a neural loss in visual performance following prolonged exposure to poor retinal image quality, thereby restricting the visual benefit of the optical correction^12^.

The elusive endpoint of subjective refraction in keratoconus is supported by a wider DOF and larger inter-subject variability in best focus in these subjects with their native HOA’s, relative to when they were reduced with RGP CL’s (Figs. 1–4 and 5A). A wider DOF indicates that a given quality of retinal image [>70% of peak IQ, in the present case (Figs. 2 and 4)] persists over a larger dioptric range and that there is little change in IQ within this dioptric space. The ability to discriminate subtle changes in IQ within this space would therefore be limited and this may account for the observed elusiveness in finding the clinical endpoint of subjective refraction in these subjects. The DOF also tends to vary inversely with peak IQ, as was observed in this (Fig. 5b) and previous studies^16,18^, suggesting that the ability of keratoconus subjects to discriminate different levels of dioptric blur also becomes better with an overall improvement in peak IQ. Better peak IQ and a narrower DOF with RGP CL may account for the observed improvement in the ability to zero-in on the endpoint of subjective refraction with RGP CL’s.

The endpoint of subjective refraction may also vary significantly when switching from spectacles to RGP CL’s, as observed in the myopic to hyperopic shift of best focus in the present study (Figs. 1–4). The best focus still remained significantly shifted away from emmetropia in keratoconic eyes (Figs. 2 and 4). This could be the result of how the keratoconic eye’s HOA’s interact with the lower-order defocus term to define IQ, as reported in previous studies on otherwise normal eyes with induced HOA’s^24^. The best focus is a rather arbitrary dioptric position within the DOF range and this could vary widely without much consequence to the underlying IQ in conditions where the DOF is wide. The myopic best focus observed in keratoconic eyes under unaided conditions therefore does not carry much value in terms of how much this specific dioptric value optimizes IQ (Fig. 1b). With a narrowing of the DOF and the through-focus curves showing a clearer peak with RGP CL’s, the best focus may indeed represent the dioptric value that best optimizes IQ (Fig. 1c). This value of best focus was observed in the hyperopic direction with RGP CL wear in these keratoconic eyes (Figs. 2 and 4).

The present results on IQ may also explain the recent psychophysical observations made in our laboratory that the binocular visual acuity of subjects with very asymmetric ectasia (reported as unilateral keratoconus in the previous study) remains unaffected and it tends to follow the monocular acuity of their fellow non-ectatic eye^8^. The binocular acuity also does not change significantly when switching from spectacles to RGP CL’s^8^. Subjects in the VAE cohort of the present study showed a clear difference in the IQ of the two eyes, with the ectatic eye’s IQ being significantly lower than that of the fellow non-ectatic eye (Figs. 3 and 4). The improvement in IQ of the keratoconic eye with RGP CL decreases the interocular difference in IQ of these subjects, albeit not to the level of age-matched controls. The binocular visual system of subjects in the VAE cohort therefore is confronted with large differences in monocular IQ’s, more so with spectacles than with RGP CL’s. Perhaps then spatial visual performance is optimized by suppressing the input from the affected eye and the stronger eye dominating the binocular visual input. Such a strategy may not be unique for keratoconus, as similar results are seen following unilateral corneal transplants and with monovision CL’s for presbyopia^25,26^. Other visual functions like stereoacuity that are critically dependent on the similarity of inputs from both eyes tends to deteriorate in the presence of an interocular difference in IQ^27,28^.

The results of this study are in agreement with the previous literature on how increases in HOA’s influence IQ in otherwise normal eyes^17,18,20,21^ or in subjects who undergo LASER refractive surgery^16^ or corneal transplants^29^. The latter two patient-based cohorts more closely resemble the present keratoconus cohort in that the increased HOA’s and the associated reduction of IQ amplitude is rather long-standing and relatively more permanent than in normal eyes with induced HOA’s. It is therefore possible that these three patient cohorts may experience some form of neural recalibration to the altered retinal IQ that may enhance or negatively impact their visual performance^12,13^. Furthermore, unlike the refractive surgery and corneal transplant cohorts where the IQ deterioration may be relatively stable post-operatively^29,30^, the keratoconus cohort may experience a progressive deterioration of IQ with worsening of the disease. It would therefore be of interest for a future study to longitudinally track changes in IQ of eyes with progressing keratoconus and to compare them to changes in psychophysical visual performance. Lastly, the present study focuses only on how IQ varies with classification of keratoconus based on spherical refractive error. In reality, keratoconic eyes experience large magnitudes of astigmatism and a similar analysis needs to be performed in the future for identifying peak IQ, best focus and DOF for combined sphero-cylindrical refraction.

This study had three limitations. First, the sample size of subjects included in the KCE and VAE cohorts of this study was relatively small and unequal. This could have partly contributed to the increased variability of the results shown in this study. A more detailed study with a larger sample size that encompass all different clinical presentations of keratoconus needs to be conducted in the future to fully understand the patterns of image quality change in this disease condition. Second, the changes in IQ of keratoconic eyes are reported here for only one design of CL correction, vis-à-vis, age-matched controls. Several advanced design CL options are currently available to manage keratoconus of different disease severities (e.g. Kerasoft CL, Rose K2 CL and Scleral CL) but the relative performance of these CL in optimizing IQ in keratoconus remains unknown^31^. The purpose of this study was to use RGP CL’s as a token measure of how optical intervention can alter the IQ in keratoconus, relative to unaided conditions and not to perform a comparative analysis of how IQ varies with disease severity or across different optical corrections. Further studies are ongoing in the laboratory to address these questions in keratoconus. Third, a minority of the subjects in the keratoconus cohort had undergone collagen crosslinking procedure for management of their disease status (Tables 1 and 2). While ideally their data should have been excluded from the study, it was decided to include them as part of the larger cohort because they met the study inclusion criteria and because the results were no different from those who did not undergo this procedure. This approach is justified because the goal of the present study was to only provide a cross-sectional view of the impact of HOA’s on the IQ of keratoconic eyes. Differences in results between those who undergo collagen crosslinking and those who do not will be revealed only in a longitudinal analysis. A future study to systematically understand longitudinal changes in IQ between optical and surgical management strategies is essential.

In conclusion, this study shows a reduction in the HOA’s of keratoconic eyes with RGP CL’s improves their optical quality, relative to unaided or spectacle wearing conditions. The residual HOA’s that remain uncorrected after RGP CL wear may explain why their optical quality remain inferior to that of age-matched controls or to that of fellow unaffected eyes. The study also shows how an improvement in the optical quality of keratoconic eyes with RGP CL’s allows better estimates of the endpoint of subjective refraction, relative to spectacles.

## Methods

The study adhered to the tenets of the Declaration of Helsinki and it was approved by the Institutional Review Board of Hyderabad Eye Research Foundation, L V Prasad Eye Institute (LVPEI). All subjects participated in the study after signing a written informed consent form. A total of 12 subjects [median (25^th^–75^th^IQR) age: 19 yrs (18–23 yrs)] with clinically diagnosed keratoconus in both eyes (KCE cohort), 9 subjects [19 yrs (18–23 yrs)] with clinically diagnosed keratoconus in one eye [very asymmetric ectasia (VAE) cohort^32^] and 20 age-matched and disease-free controls [20.5 yrs (20–21 yrs)] participated in the study. All subjects with keratoconus were recruited for the study from the cornea and contact lens services of LVPEI and all controls were recruited from within the staff and student pool of LVPEI.All subjects with keratoconus and all controls underwent a comprehensive eye examination evaluating all the aforementioned parameters before being enrolled into the study and the standard clinical management was followed for all of them, with no influence of the study on their care. The diagnosis of keratoconus was established after confirmation of clinical and topographic signs^33^. The severity of keratoconus was graded based on the Buxton *et al*. classification^34^, wherein the keratoconus was deemed as mild, if the keratometry value at the apex of the cone was ≤ 45D, moderate, if the keratometry value was in between 45D and 52D, advanced, if the keratometry value was in between 52D and 62D and, severe, if the keratometry value was > 62D.Based on this grading system^34^, both eyes of all participants in the KCE cohort and the ectatic eye of all participants in the VAE cohort were deemed to have mild to moderate levels of keratoconus. The fellow non-ectatic eye had keratometry values < 45D for all participants in the VAE cohort. All of them were experienced RGP CL wearers and they wore appropriately powered, tri-curve, back aspheric design RGP CL’s (Flouroperm 90, CLASSIC contact Lens Laboratory®, Bangalore, India) for the study. Keratoconic subjects with signs of corneal scarring, superficial punctate keratitis, frequent blinking, intolerance to RGP CL’s, monocular best-corrected high-contrast acuity worse than 20/30 with RGP CL, and any other ocular co-morbidity were excluded from the study. The left eye of one subject in the KCE cohort and the ectaticright eye of two subjects in the VAE cohort had undergone collagen crosslinking procedure more than 6-months earlier than the study recruitment time as a part of their disease management strategy. The acuity data and the corneal clarity in these subjects met the study’s inclusion criteria and hence their data were included as part of the larger cohort. All control subjects presented with monocular high-contrast acuities of 20/20 or better, keratometry values < 45D in both eyes, no history of RGP CL use and had no clinically detectable ocular pathology in their eye examination.

Wavefront aberrations of all subjects were measured for 555 nm light using the irx3™ wavefront aberrometer (Imagine Eyes®, France) with and without RGP CL in keratoconus and under unaided conditions in controls. All their eyes were cyclopleged with 2-drops of 1% Cyclopentolate Hydrochloride eye drops prior to the measurements^35^. Subjects were asked to blink before each measurement and hold their eye steady during data acquisition. Care was taken to ensure that there was no rotation or movement of CL during data acquisition. Pupil diameters were measured automatically by the software inbuilt into the aberrometer, in sync with the wavefront aberration measurements. Data was collected thrice in each eye over the entire pupil ( > 5 mm in all subjects) and averaged. The wavefront aberrations were then scaled to a constant 5 mm pupil diameter using algorithms previously developed for this purpose^36^. The wavefront aberration patterns are critically dependent on the pupil size over which they are measured and on the subject’s accommodative state^36,37^. To ensure that these parameters do not introduce an unnecessary confound on the results presented, all wavefront aberrations data were collected following cycloplegia and over a constant pupil diameter. In keratoconic eyes, wavefront aberrations data was first obtained without RGP CL’s and then with the lenses to avoid any short-term changes in corneal topography following RGP CL wear^38^. All keratoconic subjects were asked to discontinue RGP CL wear for two-weeks prior to the study visit, following the routine clinical practice pattern of the contact lens services at the study site (LVPEI).

IQ was described using the logarithm of the Neural Sharpness (logNS) metric that represents the overall effectiveness of the optical point spread function (PSF) in stimulating the neural visual system^39^. Through-focus curves was constructed for this metric for a range of target vergence (4.0D hyperopia to 4.0D myopia) by systematically changing the defocus term [Z(2,0)] in 0.5D steps while leaving the higher-order Zernike coefficients unchanged^16^. The peak of the through-focus curve and the dioptric position that corresponded to this peak represented the peak IQ and best focus, respectively^16^. The dioptric range of optical foci over which the IQ remained above 70% of peak IQ represented the DOF. The through-focus curves essentially simulated how the defocus term interacted with the HOA terms to determine the overall IQ experienced by the subject at various optical vergence states. The through-focus curves measured with the subject’s native HOA’s therefore representing a scenario where the IQ was assessed with a spectacle prescription that manipulated only the lower-order aberrations of eye, without changing HOA’s. The through-focus curves measured with RGP CL’s represented how IQ of the same subject changed when the HOA’s were reduced, relative to their native state. In the past, several cut-off values of IQ have been used to describe the DOF range (e.g. 80% of peak IQ by Sarkar *et al*.^16^ or 50% of peak IQ by Yi *et al*.^17,18^). These cut-off values are arbitrary thresholds that describe the DOF range and do not carry much clinical significance. Changing the threshold to a more conservative or liberal cut-off in this study would only result in an overall contraction or expansion of the DOF, respectively, in both unaided and RGP CL conditions without changing the relative trends between corrections. A correlation analysis was subsequently performed to determine how the best focus and the depth of focus varied with the peak IQ obtained from the through focus curves.

Statistics analyses were performed using Microsoft Excel®, SPSS® and Matlab®. The Shapiro-Wilk test indicated that most outcome variables in this study did not follow a normal distribution. Non-parametric statistics were therefore used to analyze the data reported here. The Kruskal-Wallis test was performed to analyze group-level differences in the results following by appropriate post-hoc analysis. Specifically, the Wilcoxon Sign Rank test was used to compare paired data between unaided and RGP CL wear while the Mann Whitney U test was used to compare unpaired data between keratoconics and controls. The correlation analysis was performed using Spearman’s rank correlation test. P-value < 0.05 was considered as statistically significant in this study.
